# Reactivity of Isocyanate-Functionalized Lignins: A Key Factor for the Preparation of Lignin-Based Polyurethanes

**DOI:** 10.3389/fchem.2019.00562

**Published:** 2019-08-06

**Authors:** Mareike Zieglowski, Simon Trosien, Jochen Rohrer, Sabrina Mehlhase, Simone Weber, Kerstin Bartels, Gregor Siegert, Taina Trellenkamp, Karsten Albe, Markus Biesalski

**Affiliations:** ^1^Laboratory of Macromolecular Chemistry and Paper Chemistry, Ernst-Berl Institute of Chemistry, Technische Universität Darmstadt, Darmstadt, Germany; ^2^Fachgebiet Materialmodellierung, Institute of Material Science, Technische Universität Darmstadt, Darmstadt, Germany; ^3^UPM Biochemicals, Augsburg, Germany

**Keywords:** kraft lignin, polyurethanes, polyurethane foams, isocyanates, bio-based materials

## Abstract

Using isocyanate-functionalized Kraft lignin as a reactive macromonomer for the preparation of polyurethane foams by a prepolymer technique is a well-known strategy to incorporate the biomacromolecule into a higher value polymer material. However, as of today the mechanical properties of the resulting materials are still insufficient for a number of possible applications. One reason for this limitation is that the reaction pathway and the morphological arrangement of such foams is of uttermost complexity and depends on a large number of influencing material-intrinsic factors. One important parameter is the reactivity of the functionalized lignin, which has a great impact on the interphase reaction kinetics and thus, on the geometry and mechanical properties of the resulting polyurethane foams. The reactivity is implied, amongst others, by the electron affinity of the isocyanate moiety. Herein, we investigate the reactivity of Kraft lignin modified with different commercially used isocyanates in the reaction with conventional polyols. Therefore, differently reactive prepolymers were synthesized, characterized and polyurethane foams were prepared thereof by using these compounds and the foam formation kinetics, morphological as well as mechanical properties were investigated. Finally, the results were supported by quantum mechanical calculations of the electron affinities of representative model compounds for the lignin-based prepolymers. This work gives rise to a better understanding of the effect of the reactivity and isocyanate structure linked to Kraft lignin on the polyurethane formation and enables rational choice of the isocyanate for pre-functionalization of lignin to prepare materials with better mechanical performance.

## Introduction

Polyurethanes (PUs) are a class of highly interesting materials due to their versatility that makes them valuable for various fields of day-to-day applications, such as coatings, flame retardants, insulations, adhesives, paints and foams (Chattopadhyay and Raju, 2007). Especially PU foams show unique material characteristics, such as thermal insulating, low volumetric weight and versatile mechanical properties (Singh and Jain, 2008; Yang et al., 2015; Liu et al., 2017). Chemically, polyurethanes are products of an addition reaction of a polyol and a (poly)isocyanate, respectively, and it can be considered as phase separated polymer blends which consist of alternating hard and soft segments linked to each other by different chemical as well as physical interaction means (Petrovic and Ferguson, 1991). However, the formation of such polymers is highly complex because it is influenced by a plethora of parameters. It is obvious that the molecular structure (e.g., molecular mass and geometric structure) of the single precursor components plays an important role. However, of similar importance are interactions between the individual molecules based on secondary forces (e.g., hydrogen bonds), which could influence for example the crystallinity, the phase separation, and the interphase connections of the segments, and thus, predefine the morphological structure, which greatly influences the addition reaction (Erekkath and Sreejalekshmi, 2018). For the formation of polyurethane foams, in addition to the urethane formation, a so-called blowing reaction is induced by the addition of water, which aims to generate a gas that foams the reactive mixture (most commonly CO_2_). To obtain the desired foam structure providing good material properties, urethane formation (crosslinking) of the polymer and vitrification occurs *in situ* during the blowing reaction, with an immobilized blow structure in the end. Therefore, perfectly harmonizing diffusion, reaction kinetics of the single processes (blow reaction vs. crosslinking reactions) and thermodynamic properties (e.g., glass transition/vitrification) are a crucial requirement (Delebecq et al., 2013).

To date, the primary raw materials for polyurethane production are derived from petrochemical refining of crude oil and coals (Gama et al., 2018). With increasing attention to environmental concerns about the use of fossil resources the need to find bio-based alternatives grew rapidly over the last decades (Sawpan, 2018). For this reason, much efforts were made to exploit by-products of plant materials such as lignocellulosic biomass (crops, crops residues or forestry biomass) to extract sustainable raw materials (e.g., lignin), which can be used for synthesis of the high-value PU polymer (Furtwengler and Avérous, 2018). Lignin is an amorphous, cross-linked aromatic heteropolymer which provides structural integrity and confines water transport to distinct parts of the cells in plants. Moreover, it is the second most abundant natural polymer after cellulose and accounts for nearly 30% of organic carbon in the biosphere (Boerjan et al., 2003). Fifty million tons of lignin are produced annually only in the pulp and paper industry, using different pulping processes (mainly Kraft process and sulfite process), which makes it inexpensive and readily available in bulk quantities (Türk, 2014). Although it exhibits various modification sites with different types of hydroxyl and arene moieties which are predestined to be used for the synthesis of polymeric materials (Laurichesse and Avérous, 2014), as of today only a small amount of 2 % is used to generate value-added products and chemicals (Upton and Kasko, 2016).

To incorporate lignin into polyurethane foams, up to now several approaches have been published starting from the middle of the last century (Moorer et al., 1970). Most simply, unmodified lignin was used for the foam formation and combined with an diisocyanate and an additional polyol in a single step (one-shot process) (Mahmood et al., 2016). Because the OH groups of the non-lignin polyol usually react much faster than the corresponding OHs of the lignin, a prepolymer of methylene diisocyanate (MDI) and the polyol may be formed, which would react with the lignin in a subsequent step. This sequence would greatly influence the lignin distribution. [Note that for investigation of the reaction kinetics of the polyurethane formation using unmodified lignin in the one-shot process, Barreiro and co-workers developed an elegant method. Therein, lignin was treated with MDI and a polyol [different polycaprolactones (PCL)] and the disappearance of the NCO band at ~2,270 cm^−1^ was monitored (Cateto et al., 2008, 2011)]. As a result, the obtained properties of materials made by this approach are commonly not satisfactory. One method to improve the reactivity of the lignin, is tailoring the lignin with more nucleophilic groups, e.g., via hydroxypropylation (Glasser et al., 1982; Nadji et al., 2005), modification with caprolactones (Hatakeyama et al., 2002) or introduction of additional hydroxyl groups at the arene core (Huo et al., 2012). An alternative way to gain better control of the reaction sequence and to get more efficient interphase linkage is to functionalize the lignin with a diisocyanate first and using this electrophilic precursor polymer for polyurethane foam formation (so-called prepolymer approach). The foams, which are generated by using this method, provide a significantly better performance (Chauhan et al., 2014; Gómez-Fernández et al., 2017). However, up to now, for formation of polyurethane foams with these electrophilic lignin-based prepolymers, just few studies have been published and the influence of the reactivity of the introduced isocyanate has not been investigated in detail, mainly due to non-trivial characterization of the processes and materials obtained.

Addressing the latter open questions, we were interested in investigating the influence of the molecular isocyanate structure of the corresponding lignin prepolymers on the properties of PU foams made thereof. Therefore, MDI-modified lignin (KL-MDI) as well as significantly less reactive 2,4-toluene diisocyanate (TDI)-modified lignin (KL-TDI, position 4 is bound to the lignin) and hexamethylene diisocyanate (HDI)-modified lignin (KL-HDI) providing the lowest reactivity were synthesized, characterized and incorporated into a commercial polyurethane foam formulation, respectively (for kinetic studies on the reactivity of the sole diisocyanates, see Coutinho and Rocha, 1991). The experimental results were supported by quantum mechanical calculations of the electron affinities and ionization potentials of representative model compounds. Finally, the morphology and the mechanical properties of the foams were investigated in detail to obtain better understanding of the relationship between reactivity and material properties.

## Materials and Methods

In the following section, only the most relevant materials and methods for understanding are described. A complete list of all chemicals including purity grades and suppliers of the single compounds, details about computational methods, as well as detailed information of all analytical methods used [nuclear magnetic resonance spectroscopy (NMR), infrared spectroscopy (IR), SEC (size exclusion chromatography), potentiometric titration, elemental analysis, DSC (differential scanning calorimetry), compression tests, SEM (scanning electron microscopy), and TGA (thermogravimetric analysis)] including original spectra of all synthesized compounds, further details of experimental and theoretical methods and a list of all instruments are shown in detail in Supplementary Material.

### Reagents and Materials

Kraft lignin was obtained from UPM Biochemicals (UPM Biopiva^TM^ 100, Batch 06.10.2015). All chemicals and solvents were purchased from Fisher Scientific, Sigma-Aldrich, Bernd Kraft GmbH, Tosoh, Evonic Industries AG and Grüssing and used as received unless otherwise stated. For preparation of the polyurethane foams, commercially available MDI-based isocyanate formulation (Desmodur CD-S, Covestro) and polyether-based polyol (Desmophen, 10WF15, Covestro) were used (for more details see Supplementary Material). Solvents were dried, if necessary, by using standard methods.

### Characterization of the Kraft Lignin

The used Kraft lignin was characterized via SEC, elemental analysis, DSC, IR, and ^31^P NMR for OH number determination of Argyropoulos (Granata and Argyropoulos, 1995) by using *endo*-*N*-hydroxy-5-norbornene-2,3-dicarboximide as internal standard (Zawadzki and Ragauskas, 2001). Most important material characteristics of the lignin used herein are summarized in Table 1 (for further details see Supplementary Material).

**Table 1:** Important lignin characteristics.

**Mw [g/mol]**	**Mn [g/mol]**	**Dispersity**	N content* [mol.-%]	**Hydroxy groups [mmol/g]**	**Tg [^**°**^C]**
4,780	2,075	2.30	0.26	5.90	151

*Determined via elemental analysis.

### Synthesis and Characterization of Isocyanate-Modified Lignins

#### General Protocol for Synthesizing the Prepolymers KL-MDI, KL-TDI, and KL-HDI

**Figure 1:**
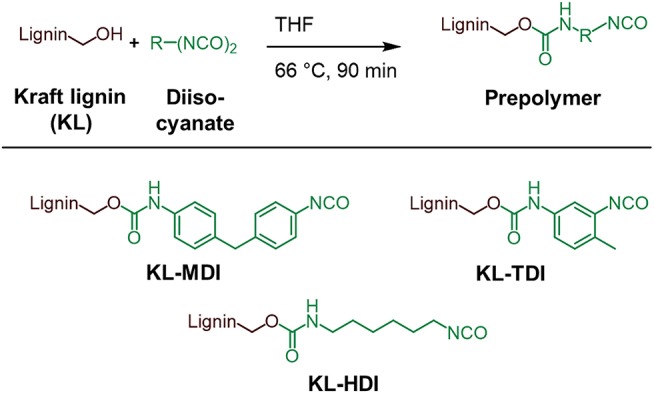
Schematic illustration of the synthesis of lignin-isocyanate prepolymers.

The synthesis of the isocyanate-modified lignins was performed according to the method of Singh and co-workers (Chauhan et al., 2014): To the desired diisocyanate (40 mmol) a solution of Kraft lignin (10 g, 59 mmol of OH groups) in dry THF (tetrahydrofuran) was added drop wise over 15–20 min at 66°C under argon atmosphere. After stirring at 66°C for 90 min the mixture was cooled to room temperature and the crude product was precipitated in 1.2 l of dry toluene. The product is centrifuged for 5 min at 4,500 rpm and the obtained supernatant is decanted. The product is washed 5 times with dry toluene (5 ×300 ml) and centrifuged at 4,500 rpm for 5 min in order to remove all non-reacted isocyanate molecules. The product was finely ground and dried at 40°C *in vacuo* overnight and stored under argon atmosphere. By using this method, the prepolymers KL-MDI, KL-TDI, and KL-HDI were synthesized and in the following further examined (Figure 1).

#### Characterization of the Isocyanate-Modified Lignins

The chemically modified lignins were characterized via elemental analysis, IR, DSC, potentiometric titrations and gravimetrical means under argon atmosphere (for details see Supplementary Material).

### Quantum Mechanical Calculations of Model Compounds of the Prepolymers

**Figure 2:**
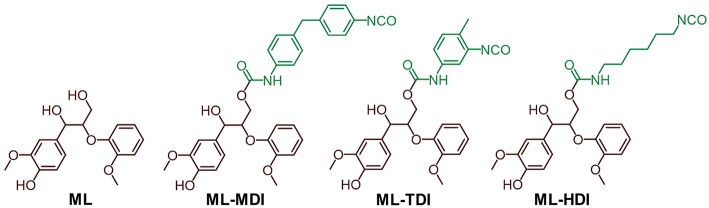
Model compounds used for DFT calculations.

Ionization potentials and electron affinities were computed using electronic structure calculations on the level of the generalized gradient approximation (GGA [REF_PBE]) of density functional theory (DFT) [REF_GPAW] by comparing charge neutral and charged systems (for details, we refer to Supplementary Material). Therefore, as model compounds the addition products of model lignin **ML** (1,3-diarylpropan-1,2-diol derivative) and the corresponding isocyanate (MDI, TDI, and HDI) were used (see Figure 2).

### Preparation and Characterization of the Lignin-Based Polyurethane Foams

#### General Protocol for Preparation of the Lignin-Based PU Foams

*Desmophen 10WF15* (178.8 g), Monoethyleneglycol (18.0 g), Teda L 33 E (2.0 g, gel catalyst), DABCO BL11 (1.0 g, 70% bis-(dimethylaminoethyl)ether and 30% dipropyleneglycol, blowing agents) and water (2.0 g, blowing agent) were mixed vigorously in a plastic beaker by using a KPG apparatus for 5 min. The resulting mixture was used as polyol component. To the polyol component (33.80 g) isocyanate-modified lignin (optimized amount of 5.7 wt.-%, 3.42 g) was added and the mixture was stirred for 10 s by using a KPG apparatus. Subsequently, the isocyanate Desmodur CD-L (22.8 g) was added. After stirring for further 10 s, the stirrer was removed. During the reaction, the start and overall foaming times were recorded.

#### Characterization of the Lignin-Based Polyurethane Foams

The foams were characterized via compression tests according to DIN standards (DIN ISO 3386-1:2015-10, 2015) (see Supplementary Material). Therefore, the foams were cut into rectangulars of 5.0 ×5.0 ×2.5 cm and compressed with a defined speed, while measuring the force and the distance. Furthermore, SEM, DSC, and gravimetric measurements were performed (for details see Supplementary Material).

## Results and Discussion

### Chemical Structure of the Functionalized Lignins

The amount of diisocyanate molecules, which have reacted with the OH groups of the lignin after transformation to the prepolymers, were determined by investigation of the nitrogen content of the compounds obtained by elemental analysis (see Table 2). In case of MDI-functionalization 1.89 mmol/g of the lignin's hydroxyl groups were modified with the diisocyanate molecules, which corresponds to a conversion of 32 mol.-% of the theoretical yield. When lignin was functionalized with TDI a lower NCO content of 1.14 mmol/g is obtained and in KL-HDI 0.97 mmol/g isocyanate moieties were linked to the lignin. This trend is caused by the fact that lignin is a highly heterogeneous biomolecule providing differently reactive hydroxyl groups. Sixteen percent of the OH groups can easily be transformed by each diisocyanate. When this amount has been abreacted, two factors are the cause that significantly more unreactive OH groups can be functionalized by using MDI than by using TDI, which both are caused by the low reactivity of the NCO group in position 2 of the TDI (Bailey et al., 1956): First, the concentration of isocyanates in solution is decreased during the reaction of the TDI, second, the reactivity of the second NCO may not be high enough to further react intramolecularly with the lignin. To investigate the impact of the latter reaction (lignin-lignin homo-crosslinking), we investigated the amount of free isocyanate groups via potentiometric titration. By this, we demonstrated that the degree of undesired crosslinking is the highest in case of synthesis of KL-MDI (free NCO = unbound NCO groups divided by amount of total diisocyanate (from elemental analysis) = 56 mol.-%, Table 2) because of the highest reactivity of the prepolymer. The reactivity of the NCO group in position 2 of TDI can be considered as low, once the other NCO group (in position 4) is abreacted. Consequently, the crosslinking of KL-TDI is negligible (free NCO = 100%, Table 2). HDI can be generally considered as unreactive and homo-crosslinking is expected to be negligible. Contrary to the expectations, a significant amount of crosslinking was determined (free NCO = 66 mol.-%) (Table 2). The reasons might be that HDI (i) provides no arene moieties, which could limit the degrees of freedom through arene-arene interactions with the lignin core and (ii) exhibits a distance and much more flexible bonds than the other isocyanates, and therefore, is capable to circumvent sterical limitations of the bulky lignin. These effects could expedite intermolecular homo-crosslinking.

**Table 2:** NCO contents of the (partially crosslinked) modified lignins by titration and elemental analysis.

**Compound**	**Total NCO amount^**a**^ [mmol/g]**	**Conversion^**c**^ [mol.-%]**	**Free NCO^**b**^ [mmol/g]**	**Free NCO [mol.-%]**	**Tg [^**°**^C]**
KL-MDI	1.89 ± 0.01	32.03 ± 0.13	1.06 ± 0.04	56	72
KL-TDI	1.14 ± 0.01	19.32 ± 0.19	1.14 ± 0.18	100	82
KL-HDI	0.97 ± 0.07	16.44 ± 1.15	0.64 ± 0.04	66	75

a *Total amount of NCO bound to the lignin determined via elemental analysis*.

b *Determined by potentiometric titration*.

c *Molar percentage of OH groups which have reacted based on the results of elemental analysis; solubility in 0.1 M NaOH solution*.

Furthermore, we were interested in the investigation of the reaction kinetics of the transformation of the synthesized lignin-based prepolymers to carbamates using a common polyol providing primary hydroxyl groups. To this end, we investigated the kinetics of the transformation of the lignin prepolymers via FTIR spectroscopy according to the method of Barreiro and co-workers (Cateto et al., 2008, 2011). To proceed according to the literature-known protocol and receive comparable results, we utilized the same polyol compound (PCL) as was used in the literature (see Supplementary Material). Interestingly, in the work of Barreiro and co-workers, the reactions between lignin, MDI and PCL are completed after about 30 min, whereas the reaction of KL-MDI takes more than 2 h. The reason might be that in the literature the reaction mixture contains small unbound MDI molecules, which could be able to generate a more flexible system so that diffusion of the polyol to the NCO plays a minor role. However, because the process is highly complex and depends on a large number of parameters, at present, the given experiments should only be discussed in qualitative manner.

### Quantum Mechanical Calculations

**Figure 3:**
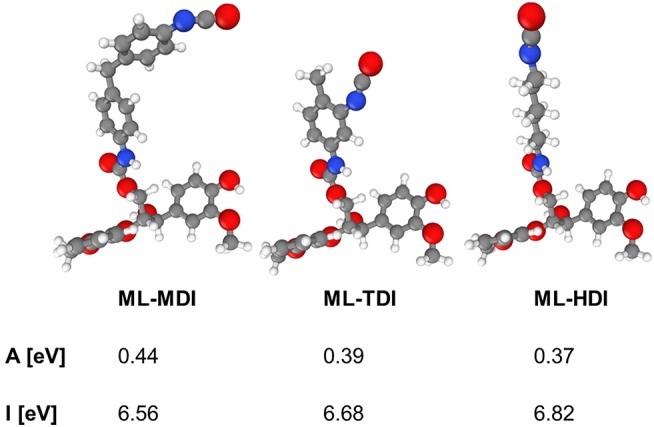
Electron affinities A and ionization potentials I for model compounds for lignin-based prepolymers determined by quantum mechanical calculations (DFT).

To support the different reactivities, we calculated the ionization potentials and the electron affinities of the model compounds ML-MDI, ML-TDI, and ML-HDI. As a result, the theoretical data correlate with our experimental findings, as expected: ML-MDI provides the highest calculated electron affinity (A_ML−MDI_ = 0.44 eV), whereas ML-TDI exhibits the second (A_ML−TDI_ = 0.39) and ML-TDI the lowest (A_ML−HDI_ = 0.37) value (Figure 3). The ionization potentials show the same tendency: I_ML−MDI_ = 6.56 eV, I_ML−TDI_ = 6.68 eV, and I_ML−HDI_ = 6.82 eV.

### Preparation and Characterization of Polyurethane Foams Using Isocyanate-Functionalized Lignins

**Figure 4:**
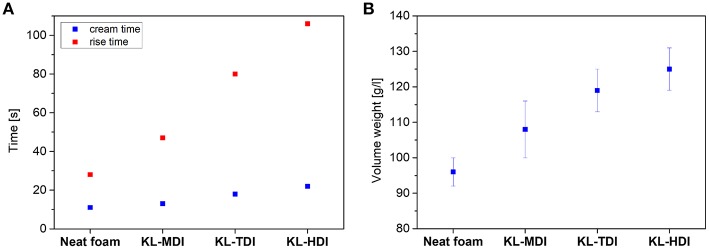
**(A)** Foaming times of different lignin-isocyanate prepolymers (cream time is the time when foaming reaction starts, rise time is the time until the volume of the foam changes); **(B)** Volume weight of polyurethane foams made of different lignin-isocyanate prepolymers.

To investigate the macroscopic effects of the different reactivities, in the next step, the synthesized isocyanate-modified lignins were used for preparation of polyurethane foams using a commercially available polyether/MDI-based formulation (for further details see Supplementary Material) and the influence on the PU foaming times were determined. In particular, the cream time (i.e., the time at which the foam begins to grow) and the rise time (i.e., the time at which the change of the foam volume stops) were analyzed (see Figure 4A). As expected, the cream and rise times of the formulations correlate with the reactivity of the isocyanate compound: the reaction of the reference foam, containing MDI as isocyanate component, is faster than the foaming process of all prepolymer-based systems: The foam formation with the MDI-modified lignin starts and stops significantly faster than the reaction of KL-TDI, respectively. KL-HDI reacts even slower, which supports the hypothesis that steric effects of the lignin molecule imply lower degrees of lignin-lignin homo coupling reactions. Caused by the slow crosslinking of KL-TDI and KL-HDI, the cell-walls of the foam ruptured before the crosslinking, and the blown structures of foams made of these compounds macroscopically collapsed before the final foam structure was set. As a result, the volume weight (density) of the KL-TDI as well as KL-HDI-based foam is distinctly higher than of the foam based on KL-MDI (see Figure 4B, for further details see Supplementary Material). However, the reference foam without any lignin provided the lowest volume weight (like also observed in the recent literature; Gómez-Fernández et al., 2017). This is caused by the fact that bubble formation, which occurs during the blow reaction, is faster than the CO_2_ generation in the lignin-based foams, which might be caused by the different viscosities of the formulations. Although it would be very interesting, determination of the viscosities during the foam formation is not trivial, due to the highly dynamic processes, low dripping times and high temperature gradients. To investigate this in more detail requires extensive method developments/adaptions and it would extend the scope of this manuscript.

Note that we have focused on the investigation of the reactivity of differently modified lignins. Since no homogeneous lignin distribution could be obtained at higher lignin contents, because of high viscosity and coagulation, we used an optimized (i.e., highest) lignin content of 5.7 wt.-% within the foam formulations. The latter was observed during empirical pre-trials (data not shown).

**Figure 5:**
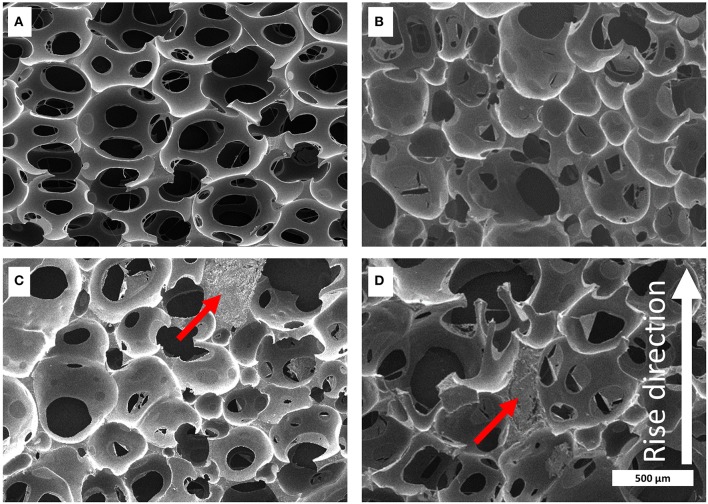
Microscopic structure of the polyurethane foams captured by SEM. Foams consisting of **(A)** neat foam, **(B)** KL-MDI, **(C)** KL-TDI, **(D)** KL-HDI. Examples of large lignin coagulates are marked with a red arrow. The foaming rise direction is shown as white arrow in **(D)** of the figure.

To understand the structure, which the foaming behavior implies in more detail, the foams were cut into small pieces and the cross-section was analyzed by scanning electron microscopy (SEM) (for further details see Supplementary Material). The lignin distribution in the foam made of KL-MDI is highly homogeneous and the foam geometry is comparable with the neat foam (Figure 5). Because of the low reactivity of KL-TDI and KL-HDI in the foams made of these prepolymers, large unreacted lignin domains (up to several hundred micrometers in diameter) were formed by phase separation, which obviously did not have reacted with the formulation and affect the foam structure. The collapse of the PU foam at the generation process causes a distinct distortion of the cell geometry, especially in case of the KL-HDI-based foam (Figure 5). Additionally, the cell size of these foams appears to be larger when compared with the material made of KL-MDI and the distribution of the cell size is much less homogeneous. The foam with KL-MDI provides cell sizes of 283 ± 8 μm diameter, whereas foams made of KL-TDI exhibits cell sizes of 394 ± 21 μm and KL-HDI 433 ± 13 μm. Note that the distorted cell structures of KL-TDI and KL-HDI limit the accuracy of the cell size determination, because of the assumption that cells are spherical. However, qualitatively the cell sizes of the latter foams are larger and the size distribution is broader.

**Figure 6:**
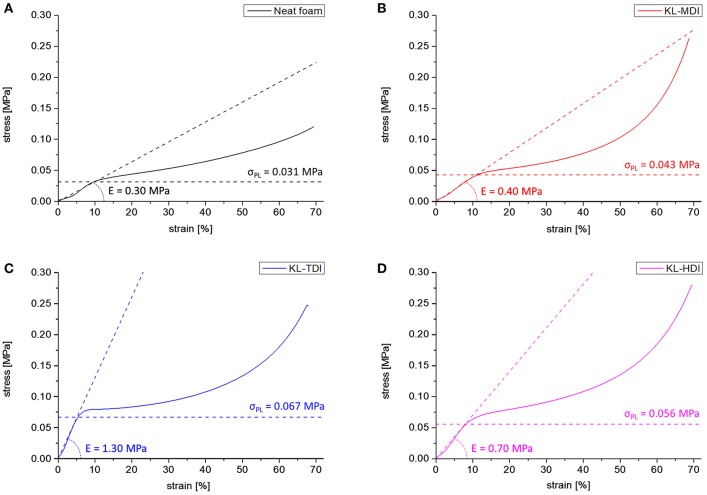
First compression curves of foams made of different lignin-based prepolymers as well as the neat foam formulation: **(A)** Neat foam, **(B)** KL-MDI-based foam, **(C)** KL-TDI-based foam, **(D)** KL-HDI-based foam. Elastic modulus of the actual elastic region as well as the proportional limit σ_PL_ are shown as dashed lines.

The foam morphologies also imply the mechanical properties. To analyze this influence in detail, samples of the foams were analyzed via compression tests according to DIN standards (EN ISO 3386), after conditioning of the samples at norm climate (23°C, 50% rel. humidity) for 2 days (see Supplementary Material). During the first deformation curve, at which first 5 to 10% of compression (decrease of the foam volume) takes place, usually the bending of cell struts occurs (actual elastic region). In this state, each lignin-based foam provides a higher elastic modulus than the neat foam (E_neat_ = 0.30 MPa) (as expected from similar lignin-based PU foams; Chauhan et al., 2014). The elastic modulus of the KL-MDI-based material is the most flexible and provides a modulus of 0.40 MPa. As expected, the foams providing an inhomogeneous lignin distribution (foams made of KL-TDI and KL-HDI) are distinctly less flexible (E_KL−TDI_ = 1.30 and E_KL−HDI_ = 0.70 MPa) (Figure 6). This is caused by the coagulated lignin domains, which may provide a reinforcement effect (Figure 5). The fact that the flexibility of the KL-TDI-based foam is lower than the KL-HDI-based foam can be explained by the strongly ruptured cell geometry of the latter materials.

**Figure 7:**
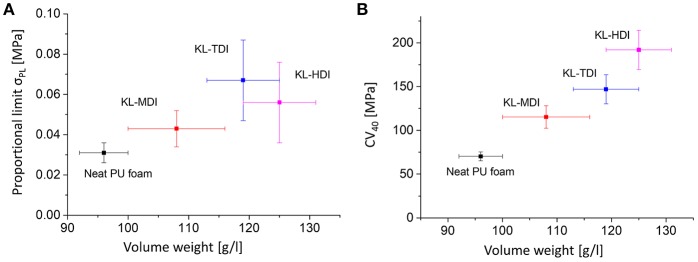
**(A)** Proportional limit σ_PL_ of the different foams vs. the volume weight. **(B)** CV_40_ values of the different foams vs. the volume weight.

As a result of the lignin-reinforcement and the ruptured cell geometry, the compressive strength of the initial elastic region (proportional limit σ_PL_) correlates not linearly with the volume weight (see Figure 7A). This is particularly noticeable in case of the KL-HDI-based foam, which provides the highest volume weight but not the highest proportional limit: σ_PL,KL−HDI_ = 0.056 MPa and σ_PL,KL−TDI_ = 0.067 MPa, while the volume weights are δ_KL−TDI_ = 115 and δ_KL−HDI_ = 125 g/l. Once the foams have been compressed, the effects of the cell geometry and reinforcement of the material by lignin domains are extinguished. Consequently, if the so-called plateau stress of the materials is compared according to DIN standards at the 4th compression cycle (i.e., the CV_40_ value = the force which is needed to compress the foam volume to 40%), the results clearly correlate with the volume weights (see Figure 7B): For compression of the neat foam, providing the lowest volume weight, the lowest force is needed (CV_40,neat_ = 70.1 N). The lignin-based foam with the lowest volume weight containing KL-MDI possesses the lowest CV_40_ value for the foams obtained from lignin prepolymers (CV_40,KL−MDI_ = 115.3 N), whereas the KL-HDI-based foam needs most force to be compressed to 40% (CV_40,KL−HDI_ = 192.0 N; in between the value of the KL-TDI-based foam: CV_40,KL−TDI_ = 147.0 N) (see Figure 7B).

In the final stages of deformation, densification takes place. At this point the stress rises steeply and the cell walls are finally crushed (Ashby, 2006), which leads to an irreversible deformation of the material. In particular, 4.3% of the foam made of KL-MDI were irreversibly deformed, in case of KL-TDI 5.0%, and in case of the KL-HDI-based foam 6.9% (complete mechanical testing curves including hysteresis curves are shown in Supplementary Material), respectively.

## Conclusion

In conclusion, we prepared different lignin-based isocyanates by treatment of Kraft lignin with various diisocyanates and analyzed the impact of the molecular structure of the diisocyanate moieties on the reactivity of the resulting prepolymers. To this end, different macromonomers were synthesized by treatment of Kraft lignin with different diisocyanates and fully characterized. As a result, the structure of the different prepolymer differs vastly with regard to the extent of intra- and intermolecular lignin-lignin homo coupling (i.e., degree of crosslinking/amount of reactive NCO groups), which occurs during the synthesis of the compounds, implied by the reactivity of the NCO moieties. The results are in good agreement with the electron affinity and the ionization potential, which were determined for representative model compounds of the prepolymers. Furthermore, the compounds were used for preparation of PU foams and the influence of the reactivity on their macroscopic properties was determined. By this, the relationship between molecular structure, foaming kinetics, volume weight, lignin distribution, cell geometry and mechanical properties of the resulting materials was investigated. As expected, the foaming times and the density of the foams correlate with the reactivity of the utilized lignin prepolymer. Furthermore, the morphological as well as the mechanical properties of the different materials differ distinctly because of the diverging kinetics of the interphase reactions. This work gives rise to a better understanding of the relationship between molecular structure and macroscopic properties of lignin-based polyurethane foams and therefore, enables the rational preparation of foams with tailor-made macroscopic properties.

## Data Availability

All datasets generated for this study are included in the manuscript and/or the Supplementary Files.

## Author Contributions

All authors listed, have made substantial, direct, and intellectual contribution to the work. MZ, SW, KB, and GS performed the experiments and characterized the materials. SM, ST, and MZ analyzed the data, reviewed the literature, and wrote the manuscript. JR and KA coordinated and performed the quantum mechanical calculations. MB and ST set-up and coordinated the project. All authors discussed and reviewed the results. All authors discussed, reviewed, and approved the manuscript.

### Conflict of Interest Statement

The authors declare that the research was conducted in the absence of any commercial or financial relationships that could be construed as a potential conflict of interest.
