# Supplementary material for: Reactivity of Isocyanate-Functionalized Lignins: A Key Factor for the Preparation of Lignin-Based Polyurethanes
Source: Front Chem. 2019 Aug 6;7:562. doi: 10.3389/fchem.2019.00562 (PMC6691062; doi:10.3389/fchem.2019.00562)
Supplement: Supplementary file 1 [file Data_Sheet_1.PDF]

Supporting Information

Reactivity of Isocyanate-functionalized Lignins: A Key Factor for the Preparation of Lignin-based Polyurethanes

*Mareike Zieglowski,[1] Simon Trosien,[1] Sabrina Mehlhase,[1] Simone Weber,[1] Kerstin Bartels,[1] Gregor Siegert,[1] Taina Trellenkamp,[2] and Markus Biesalski[1]**

[1]Laboratory of Macromolecular Chemistry and Paper Chemistry, Ernst-Berl Institute of Chemistry, Department of Chemistry, Technische Universität Darmstadt, Germany

[2]UPM Biochemicals, Augsburg, Germany

Content

1. Instruments and materials S-2
   a. Substances S-2
   b. Instruments and characterization methods S-2
   c. Lignin characterization S-5
2. Synthesis and Production Procedures S-8
   a. Isocyanate modification protocols S-8
   b. Production of foams S-8
3. Quantum mechanical calculations S-9
4. Kinetics measurements S-11
5. Mechanical characterization: compression tests S-12
6. Spectra S-14
   a. DSC thermograms S-14
   b. IR spectra S-17
   c. Compression test S-18
7. References S-20

1. Instruments and materials

a. Substances

Chemical	Supplier
Lignin BIOPIVA 100	UPM GmbH
Methylenediphenyl diisocyanate (Desmodur CD-S)	Covestro AG
Toluene-2,4-diisocyanate diisocyanate	Sigma Aldrich
Hexamethylene diisocyanate	Acros Organics
Tetrahydrofuran (99.9%)	Acros Organics
Desmophen 10WF15	Covestro AG
Monoethylenglycol	Grüssing GmbH
TEDA L33E	Tosoh
DABCO BL11	Evonik Industries AG
Dibutylamine (2 M in toluene)	Bernd Kraft GmbH
Hydrochloric acid (1 M)	Grüssing GmbH
Polycaprolactone (M_n≈530)	Sigma Aldrich

b. Instruments and characterization methods

Climate conditioning of the samples

For climate conditioning all samples were stored for at least 2 days in a standard climate room (23 °C, 50% rel. humidity) before measurement.

Centrifugation

Centrifugation was performed using a *Thermo Scientific Hereaus Megafuge 8* centrifuge.

Compression tests

Compression tests were carried out on a *Zwick/Roell zwickiLine Z1.0 TN* according to the DIN EN ISO 3386-1.(DIN ISO 3386-1:2015-10, 2015) The software *testXpert II* was used.

Density determination

The density is determined gravimetrically. For this the foams are cut into a size of 5x5x2.5 cm and weighed by using an analytical balance (Typ: CP64-OCE from Sartorius).

Differential scanning calorimetry

The DSC measurements are performed with a DSC 1 Star^e System from Mettler Toledo. The measurements were carried out under nitrogen atmosphere with a heating rate of 10 K/min in a temperature range from −100 °C to 150 °C.

Elemental analysis

Elemental analysis was carried out on a *Elementar VarioEL III CHN* with TCD and helium as carrier gas and oxygen as combustion gas.

Size exclusion chromatography (SEC)

The SEC measurements were performed on a 1260 Infinity GPC/SEC system from Agilent Technologies. The column used is of the MCX type, with a column dimension of 8–300 mm, a particle size of 5 µm, a porosity of 100–1000 Å and a molecular weight range of 100–70k Da. The concentration of the samples was between 1–3 mg/ml with a flow rate of 1 ml/min. A UV or RID detector was used. Aqueous NaOH (0.1 M) was used as eluent and the calibration was performed against polystyrene sulfonate.

IR spectroscopy

Fourier transform infrared (FT-IR) spectroscopy was performed on a *PerkinElmer Instruments Systems One FT-IR Spectrometer*, coupled to a *universal attenuated total reflectance* (UATR) unit. Usually ten scans are registered per spectrum with a resolution of 4 cm^{-1}, between wavenumbers of 4 000 cm^{-1} and 650 cm^{-1}. The software *Spectrum Version 6.3.5.0177 Copyright® 2009 PerkinElmer Inc.* was used.

NMR spectroscopy

NMR spectroscopy was performed on a *Bruker AC300* (300 MHz) and *DRX500* (500 MHz) spectrometers at 25 °C. Chemical shifts are shown in δ units, parts per million (ppm).

Proton signals are relative to tetramethylsilane (TMS) with δ = 0 ppm and are calibrated by reference to residual protons in the deuterated solvents ($CHCl_3$ = 7.26 ppm,). The software *MestReNova Version 11.0 (Mestrelab Research S.L.)* was used.

^{31}P NMR was performed by using the method of Argyropoulos in DMF/$CDCl_3$ (Granata and Argyropoulos, 1995). Signals are calibrated by reference to phospitylated *endo-N*-hydroxy-5-norbornene-2,3-dicarboximide (δ = 151.9 ppm) used as internal standard (Zawadzki and Ragauskas, 2001).

Potentiometric titration

The measurements are performed with the *905 Titrando* instrument from *Metrohm* and the associated *tiamo 2.3* software. The data obtained is evaluated with the *OriginPro 9.0.0* software.

Isocyanate content was determined with potentiometric titration according to DIN EN ISO 14896 (DIN ISO 14896:2009, 2009). A sample of 3 g isocyanate modified lignin in 25 ml THF was stirred until dissolved. A reaction solution of dibutyl amine / isocyanate solution (DBA, 2 M in toluene) was added followed by the addition of isopropanol to increase the titration volume after 10-15 minutes. Potentiometric titration was performed using hydrochloric acid (1 M) determining the excess (unreacted) DBA. Inflection points were determined using a Double Boltzmann sigmoidal fit in Origin Lab 8.6. Each sample was investigated three times. Blank measurements without sample were carried out with bromophenol blue as indicator. Isocyanate content %NCO was calculated with the volume of HCl during blank measurement V_{Blank}, the volume of HCl used during Lignin-NCO titration $V_{Lignin\text{-}NCO}$ and $m_{Lignin\text{-}NCO}$ the weight of the sample:

$$\%NCO = 4.2 \cdot \frac{V(blank) - V(Lignin-NCO)}{m(Lignin-NCO)}$$

Isocyanate content y_{NCO} in mmol NCO per g sample was calculated according to DIN EN ISO 14896.(V., 2009)

$$y_{NCO} = \frac{\%NCO}{4.2}.$$

Modification rates f_{NCO} can be calculated using the following equation:

$$f_{NCO} = \frac{y_{NCO}}{Total-OH} \cdot 100\% \,.$$

Scanning electron microscope

For Scanning electron microscopy (SEM) a *FEI/Philips XL30 FEG* (Philips, Amsterdam, Netherlands) with accelerating voltage of 10 kV was used. The SEM samples were coated with 10 nm platinum/palladium (80%/ 20%) with a sputter coater *208 HR* from *Cressington*.

Thermogravimetric analysis

The TGA measurements were carried out on a *TGA 1* from *Mettler Toledo*. A heating rate of 10 K/min in a temperature range of 30 °C to 600 °C was measured. First of all, the samples are kept in a nitrogen atmosphere during heating, after they have been measured at 600 °C for a further 60 minutes, an air atmosphere is selected for a further 60 minutes.

c. Lignin characterization

Molecular weight: M_n = 2075 g/mol; M_w = 4780 g/mol

Dry content: 99%

Lignin spectra: IR and ^{1}H NMR spectra are shown in Figure S-1 and Figure S-2.

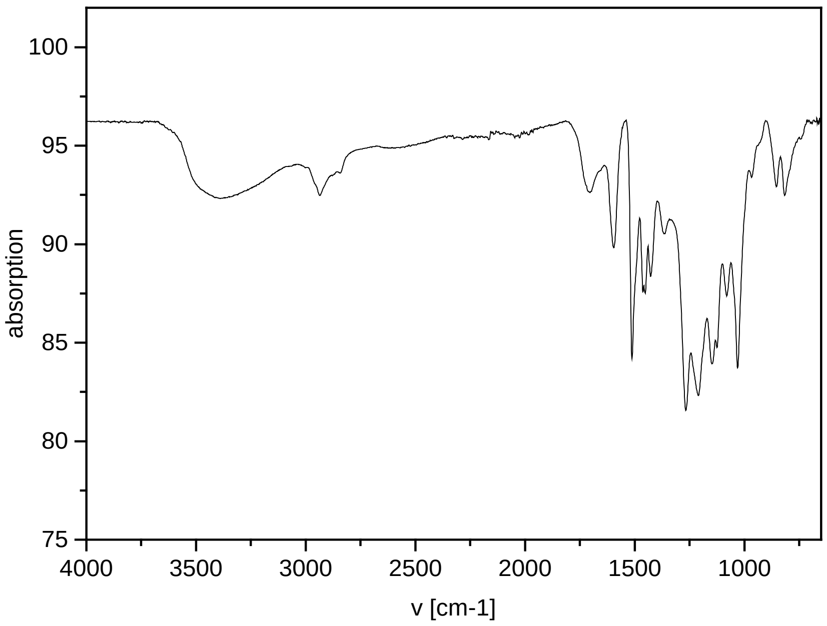

Figure S-1. IR spectrum of Domtar Kraft lignin.

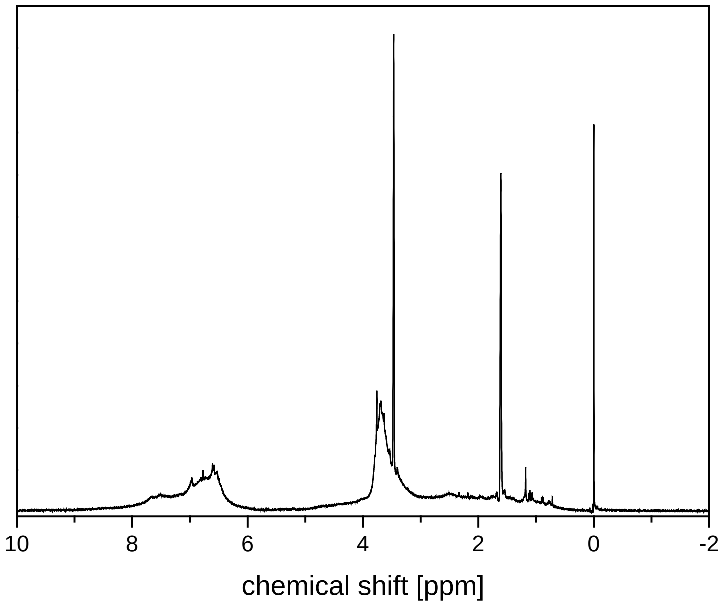

Figure S-2: ^{1}H NMR spectrum of Domtar Kraft lignin.

DSC

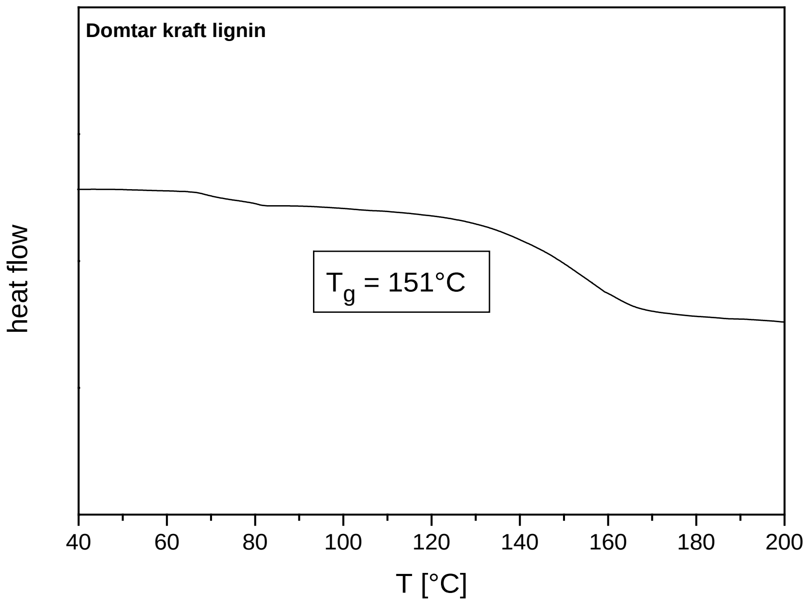

Figure S-3: DSC thermogram of Domtar Kraft lignin.

TGA

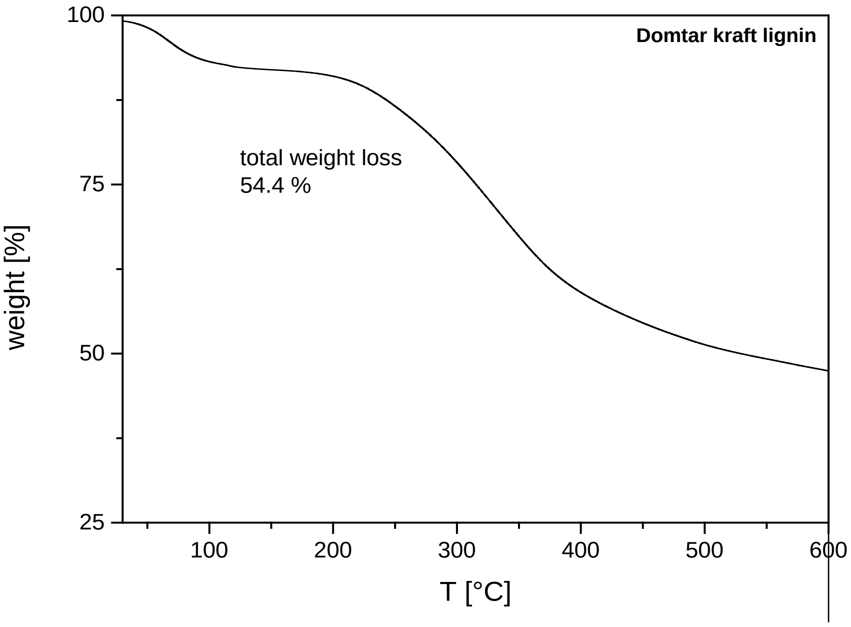

Figure S-4: TGA measurement of Domtar Kraft lignin.

OH groups.

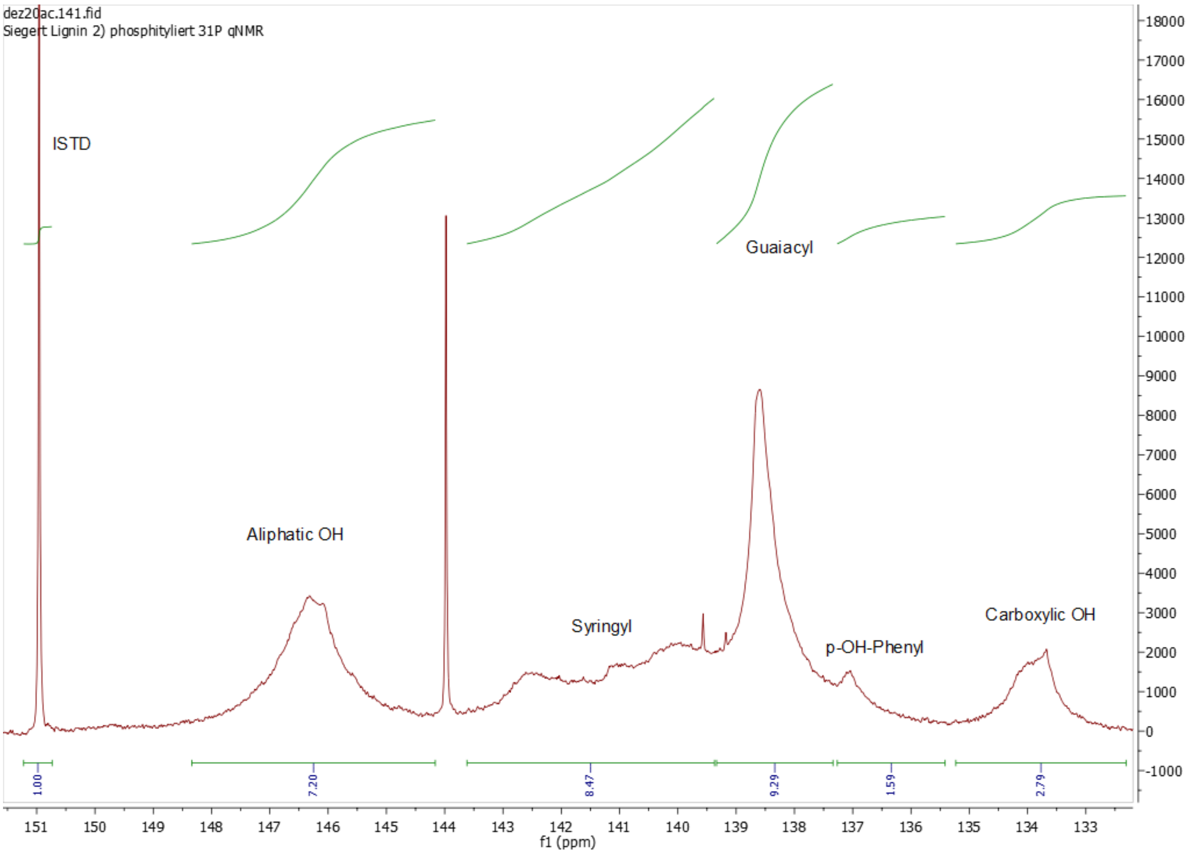

Sample	Aliphatic OH [mmol/g]	Syringyl OH [mmol/g]	Guaiacyl OH [mmol/g]	*p*-OH-phenyl [mmol/g]	Carboxylic-OH [mmol/g]	**Total OH [mmol/g]**
1	1,66	1,77	1,65	0,31	0,55	**5,94**
2	1,44	1,69	1,86	0,32	0,56	**5,87**

Figure S-5. ^{31}P NMR spectrum of phosphitylated Kraft lignin (top); content of different types of OH groups in mmol/g (bottom)

2. Synthesis and Production Procedures

a. Isocyanate modification protocols

General protocol for synthesis of isocyanate modified lignins

To a diisocyanate (1.5 eq, under argon atmosphere) a solution of lignin (1 eq in THF) was added drop wise over 15-20 minutes at 90 °C. After stirring at 90 °C for 90 minutes the solvent was cooled and precipitated in 1.2 L of toluene. The product is centrifuged for 5 min at 4500 rpm and the obtained supernatant is decanted. The product is washed 5 times with toluene and centrifuged at 4500 rpm for 5 minutes. The product was finely ground and dried at 40 °C *in vacuo* overnight.

DSC and IR spectra are shown in section **4. Spectra**.

Synthesis of methylenediphenyl diisocyanate-functionalized lignin (KL-MDI)

Dissolve 10 g (52 mmol OH, 1 eq) Kraft lignin in 150 ml dry THF and react with 75 g (77 mmol, 1.5 eq) methylenediphenyl diisocyanate (**MDI**) to yield 6.23 g of KL-MDI providing 1.06 mmol/g of free NCO groups. Elemental analysis: C: 64.68%, H: 4.98%, N: 5.55%

Synthesis of toluene-2,4-diisocyanate diisocyanate-functionalized lignin (KL-TDI)

Dissolve 20 g (105 mmol OH, 1 eq) Kraft lignin in 300 ml dry THF and react with 28 g (174 mmol, 1.5 eq) toluene-2,4-diisocyanate diisocyanate (**TDI**) to yield 12.05 g of **KL-TDI** providing 1.14 mmol/g of free NCO groups. Elemental analysis: C: 61.87%, H: 5.26%, N: 3.43%

Synthesis of hexamethylene diisocyanate-functionalized lignin (KL-HDI)

Dissolve 20.01 g (104.83 mmol OH, 1 eq) Kraft lignin in 300 ml dry THF and react with 27 g (161mmol, 1.5 equq) hexamethylene diisocyanate (**HDI**) to yield 13,34 g of **KL-HDI** providing 0.64 mmol/g of free NCO groups. Elemental analysis: C: 61.58%, H: 5.94%, N: 2.55%

b. Production of foams

Desmophen 10WF15 (178.8 g), Monoethyleneglycol (18.0 g), Teda L 33 E (2.0 g, gel catalyst), DABCO BL11 (1.0 g, 70% bis-(dimethylaminoethyl)ether and 30% dipropyleneglycol, blowing agents) and water (2.0 g, blowing agent) were mixed vigorously in a plastic beaker by using a KPG apparatus for 5 minutes. The resulting mixture was used as polyol component. To the polyol component (33.80 g) (isocyanate-modified) lignin (5.7 wt.-%, 3.42 g) was added and the mixture was stirred for 10 s by using a KPG apparatus. Subsequently, the isocyanate Desmodur CD-L (22.8 g) was added. After stirring for 10 s, the stirrer was removed. Shortly after the foaming process starts. During the reaction, the start and overall foaming time were recorded.

3. Quantum mechanical calculations

All calculations were performed on the basis of density functional theory using the GPAW code (Mortensen et al. 2005). For exchange and correlation (XC), we compared the standard PBE (Perdew et al. 1996) and the two hybrid functionals PBE0 (Adamo 1999) and B3LYP (Stephens et al. 1994). The spin-polarized Kohn-Sham equations are solved at the Γ-point using finite differences with a grid spacing of h = 0.14 Å and open boundary conditions. A Fermi-Dirac smearing with initial width of w = 0.1 is employed and then reduced to the w = 0 case (step function). The molecules are generated using ChemDraw and then structurally optimized until forces on individual atoms do not exceed 0.05 eV/Å. For these optimizations the quasi-Newton method implemented in the atomic simulation environment (ase) package is used (Larsen et al. 2017). For each atom/molecule three sets of calculations are performed: a charge-neutral calculation, a calculation with one additional electron and a calculation with one electron removed. The correspontING total energies are denoted as E_0, E_- and E_+. From these energies we obtain the ionization potential (I) and the electron affinity (A) as

$$I = E_+ - E_0$$

$$A = E_0 - E_-$$

As shown in Figure S-6 and Table S-1, there is a reasonable agreement in the hierarchy and absolute values of these quantities for various elements and small molecules, irrespective of the choice of the XC functional, showing that the above procedure results in reliably calculated values of I and A. Therefore, calculations for KL-MDI, KL-HDI and KL-TDI are exclusively performed using the PBE functional. For these larger molecules, electronic convergence is hard to achieve using a Fermi-Dirac smearing with zero width, especially upon charging. Due to this, a smearing width of 0.01 was used.

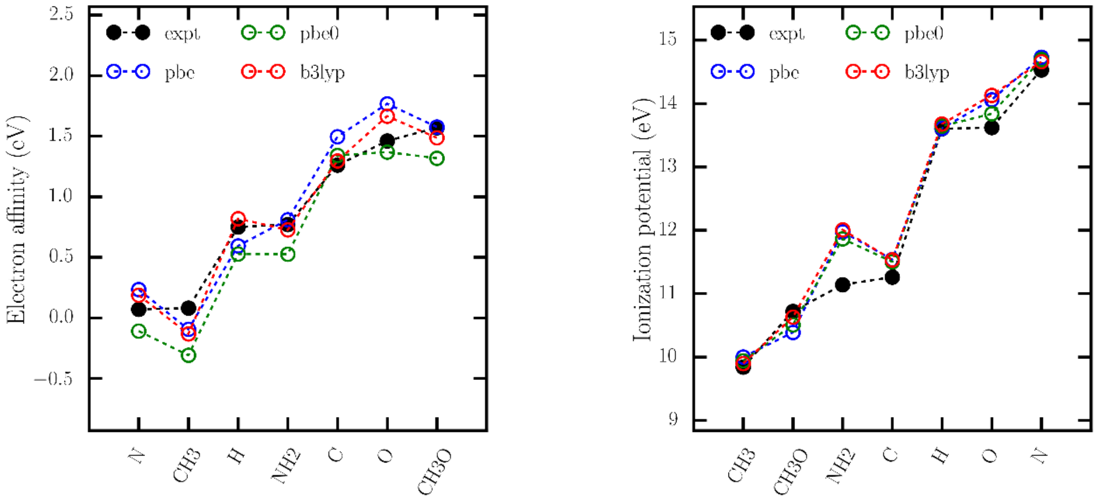

Figure S-6. Comparison of electron affinity (left) and ionization potential (right) of different species determined using different functionals to each other and with the experimental value (expt).

Table S-1. Ionization potential and electron affinity of different species calculated by using different functionals (PBE, PBE0 and B3LYP) as well as the corresponding experimental values (Expt).

	Expt		PBE		PBE0		B3LYP	
	I [eV]	A [eV]	I [eV]	A [eV]	I [eV]	A [eV]	I [eV]	A [eV]
H	13.60	0.75	13.61	0.59	13.64	0.53	13.67	0.82
C	11.26	1.26	11.53	1.50	11.50	1.34	11.53	1.30
N	14.53	0.07	14.73	0.23	14.69	−0.11	14.66	0.19
O	13.62	1.46	14.06	1.77	13.84	1.37	14.12	1.67
CH_3	9.84	0.08	9.99	−0.09	9.93	−0.31	9.89	−0.13
CH_3O	10.72	1.57	10.38	1.58	10.51	1.32	10.63	1.48
NH_2	11.14	0.77	11.97	0.81	11.86	0.53	12.00	0.73

4. Kinetics measurements

For investigation of the reaction kinetics of the prepolymers, we treated the functionalized lignin with a polycaprolactone (PCL530) and monitored the reaction progress by FTIR-ATR. To this end, a mixture of PCL (5,4 g, 10,18 mmol, 200 mg KOH/g), the desired lignin prepolymer (0,4 mmol) and THF (30 ml) was stirred at 66 °C under argon atmosphere.

KL-MDI

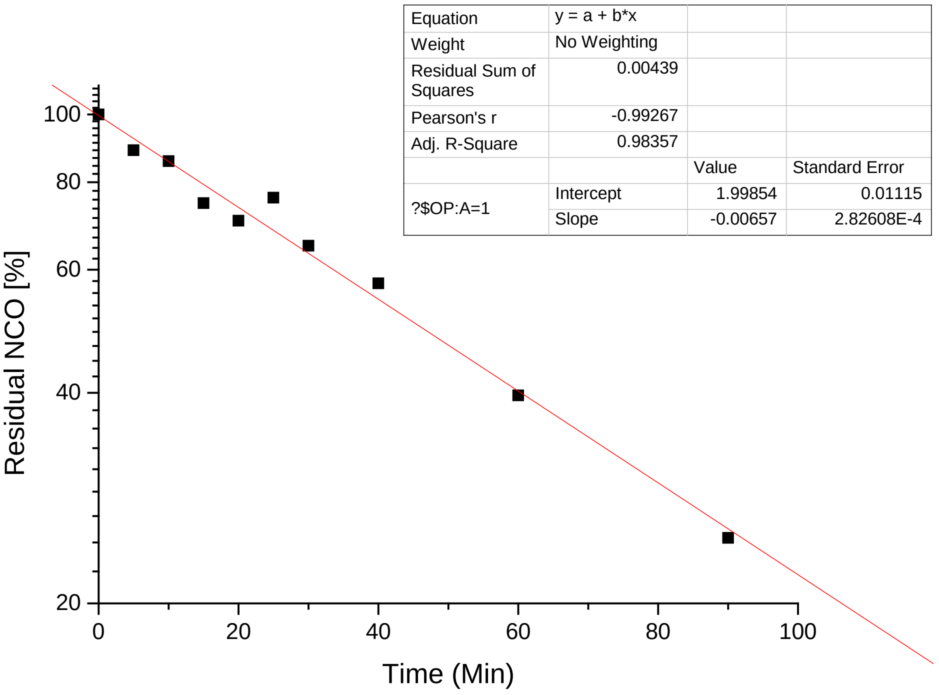

KL-HDI

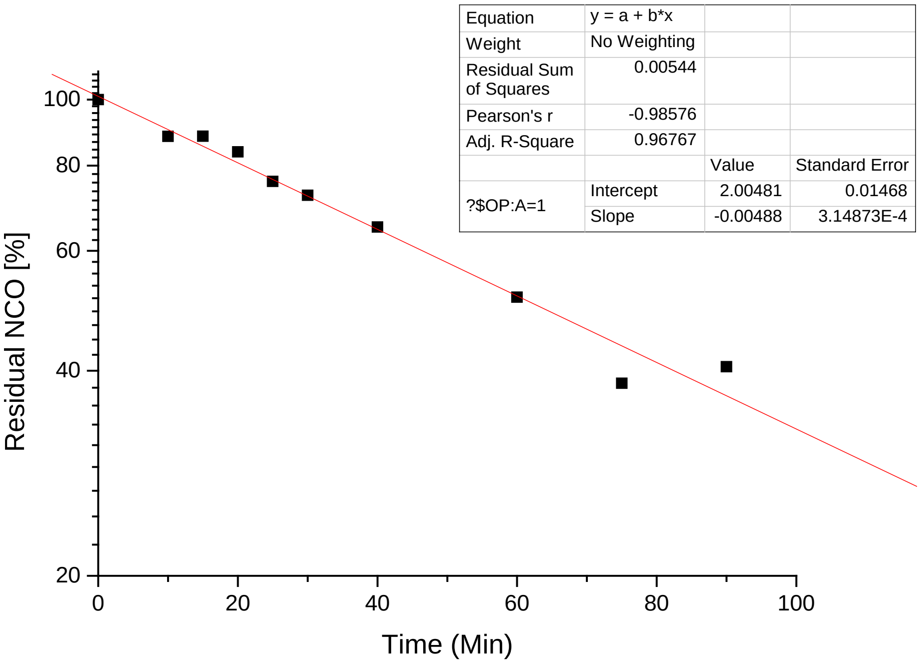

5. Mechanical characterization: compression tests

Measurements of test specimens were performed according to DIN EN ISO 3386-1.

The specimens were cut into pieces of about 25×50×50 mm and compressed between a support surface and a pressure plate at a uniform relative vertical loading rate of 100 mm/min. The resulting data (exact height of the test specimen and compressive stress value) are summarized in Table S-2. The original spectra are shown in section 4 of the Supporting Information (Spectra).

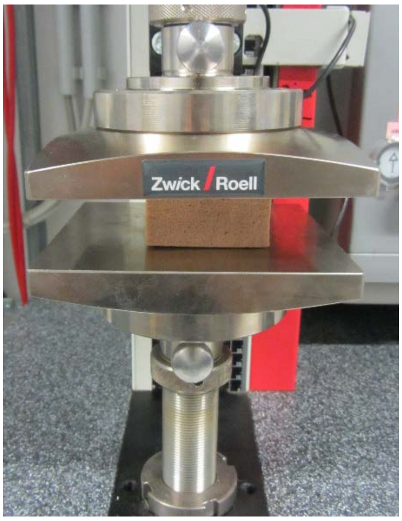

Figure S-7. Setup for deflection tests according to DIN EN ISO 3386-1.

Table S-2. Results of compression tests of polyurethane foams made of different lignins.

**Lignin derivative**	**h_0 [mm]**	**CV_{40} [N]**
None/neat resin	22.7 ± 0.4	70.1 ± 5.2
Lignin-MDI (5.7 wt.-%)	26.3 ± 1.7	115.3 ± 12.8
Lignin-TDI (5.7 wt.-%)	23.5 ± 0.9	147.0 ± 16.6
Lignin-HDI (5.7 wt.-%)	23.7 ± 0.6	192.0 ± 22.49

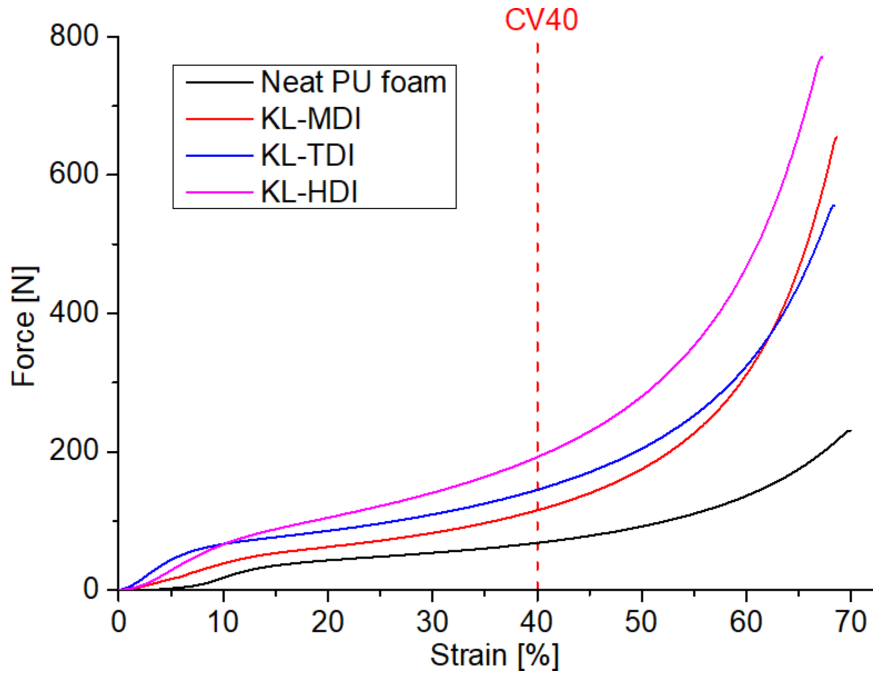
CV40
Neat PU foam
KL-MDI
KL-TDI
KL-HDI
Force [N]
Strain [%]
0
200
400
600
800
0
10
20
30
40
50
60
70

6. Spectra

a. DSC thermograms

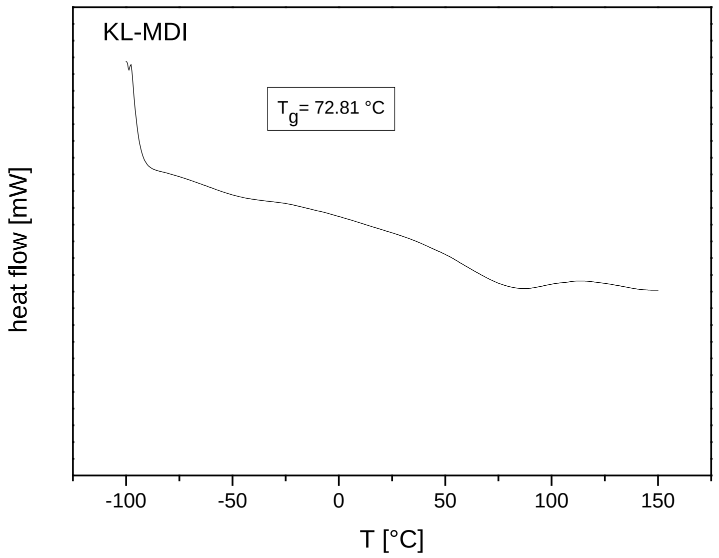

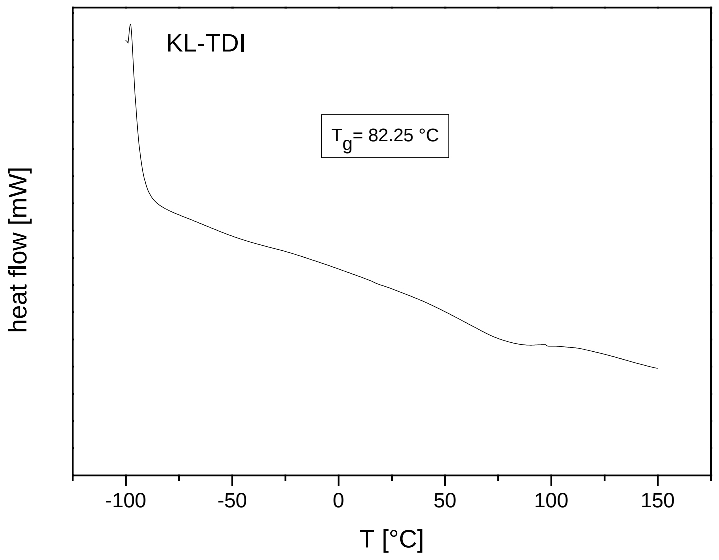

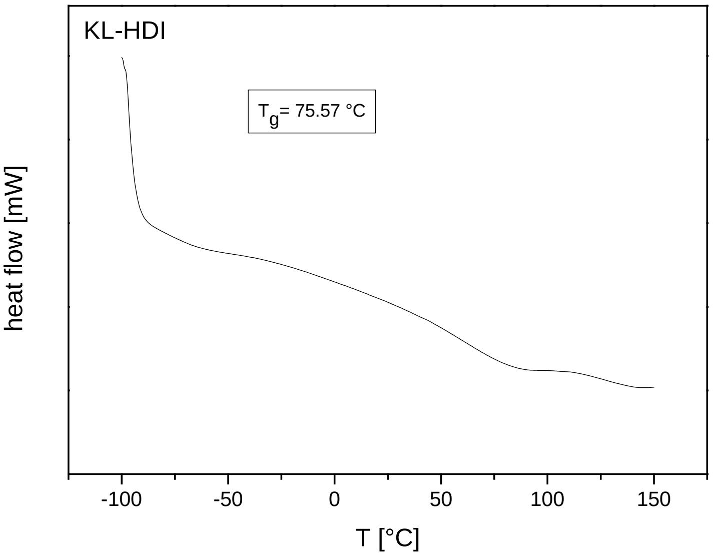
KL-HDI
T_g= 75.57 °C
heat flow [mW]
-100
-50
0
50
100
150
T [°C]

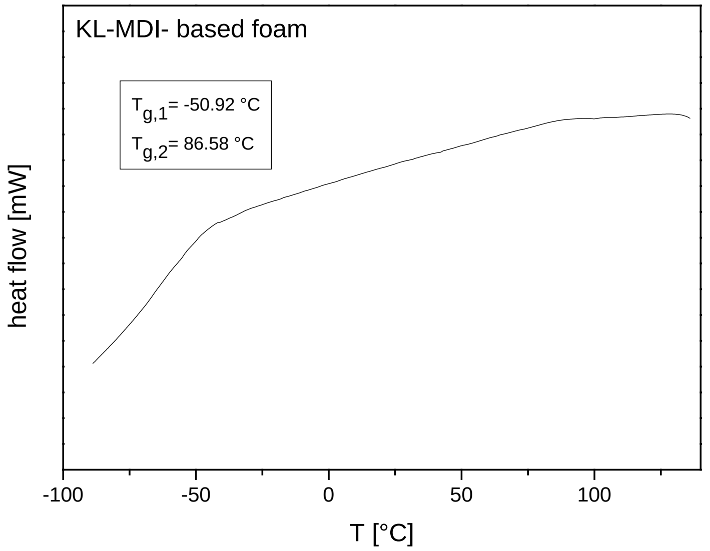
KL-MDI- based foam
$T_{g,1}$= -50.92 °C
$T_{g,2}$= 86.58 °C
heat flow [mW]
-100
-50
0
50
100
T [°C]

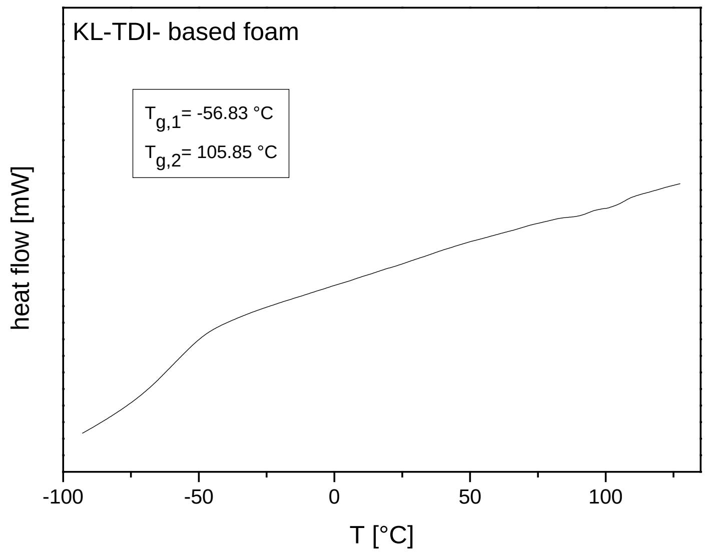
KL-TDI- based foam
Tg,1= -56.83 °C
Tg,2= 105.85 °C
heat flow [mW]
-100
-50
0
50
100
T [°C]

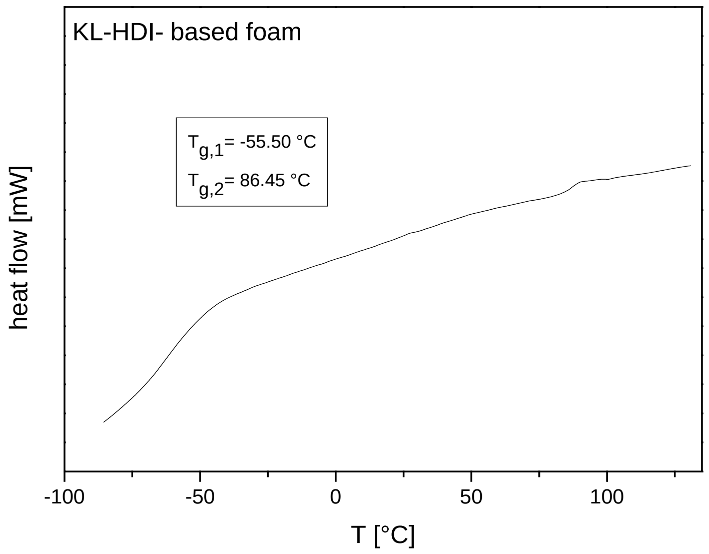
KL-HDI- based foam
Tg,1= -55.50 °C
Tg,2= 86.45 °C
heat flow [mW]
-100
-50
0
50
100
T [°C]

b. IR spectra

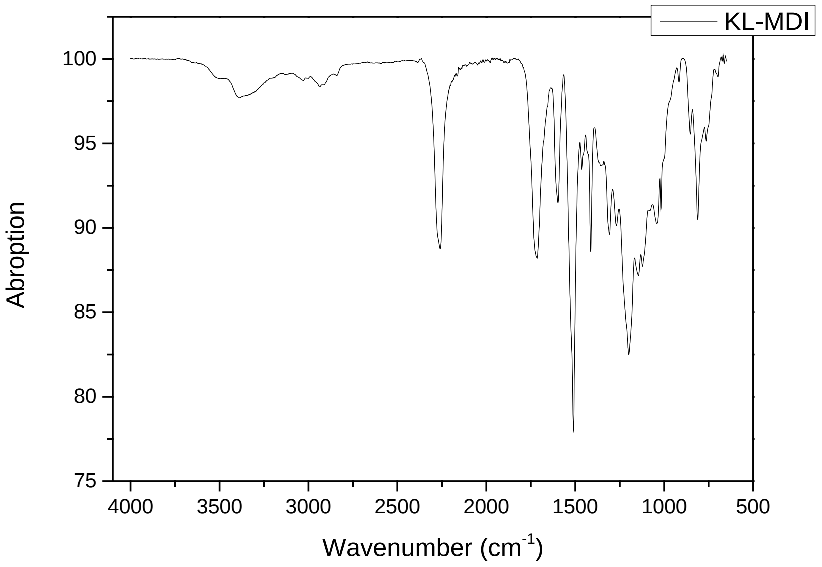
KL-MDI
Abroption
100
95
90
85
80
75
4000
3500
3000
2500
2000
1500
1000
500
Wavenumber (cm-1)

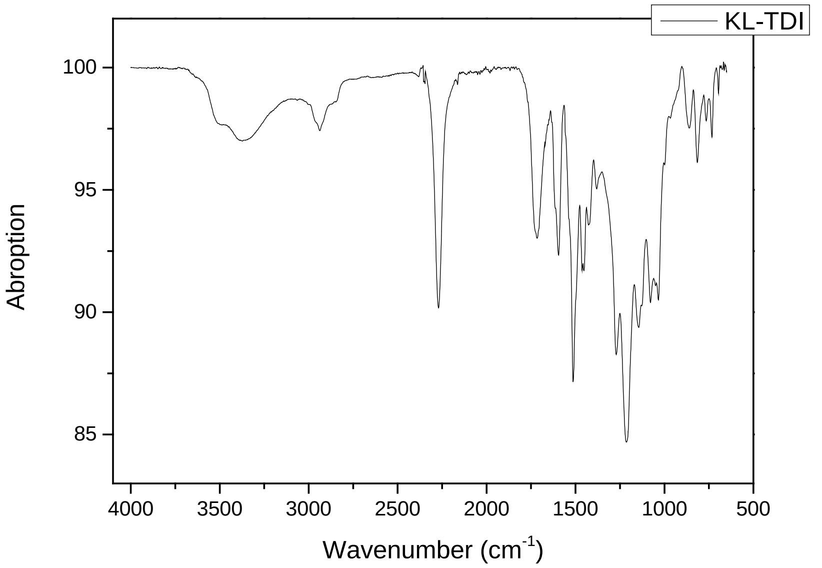
KL-TDI
Abroption
100
95
90
85
4000
3500
3000
2500
2000
1500
1000
500
Wavenumber (cm-1)

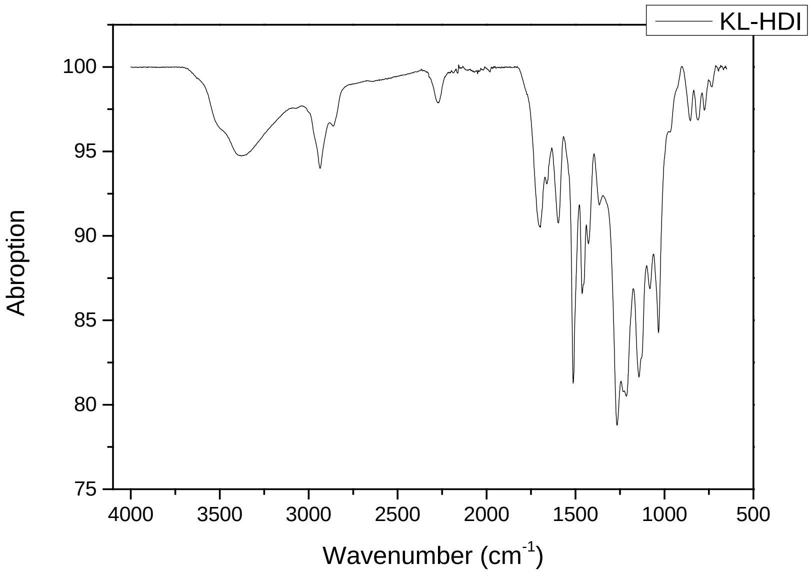

c. Compression test

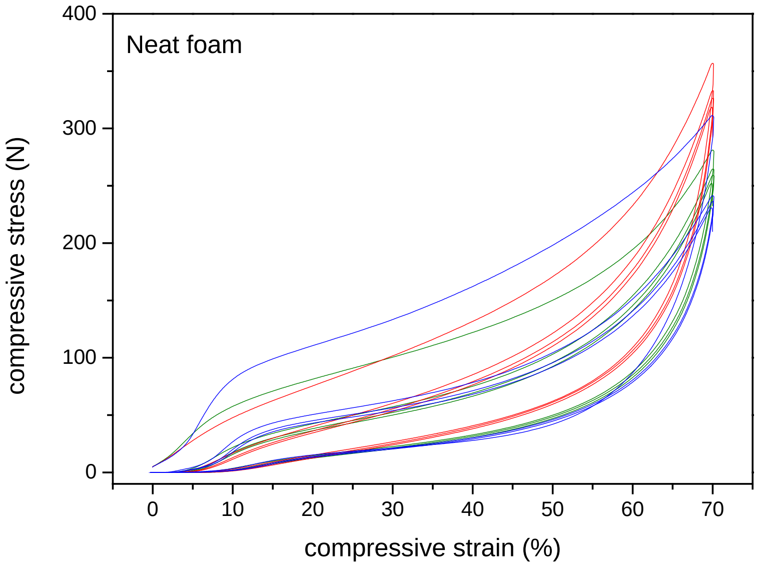

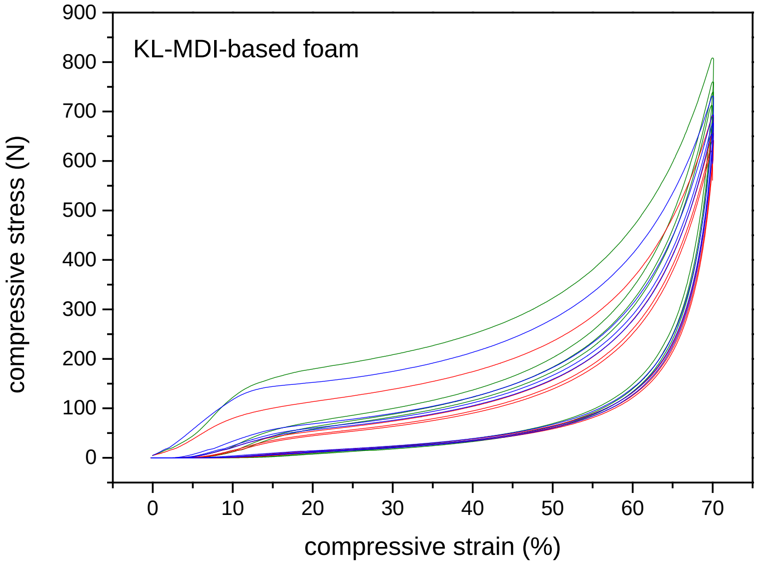
KL-MDI-based foam
compressive stress (N)
compressive strain (%)
900
800
700
600
500
400
300
200
100
0
0
10
20
30
40
50
60
70

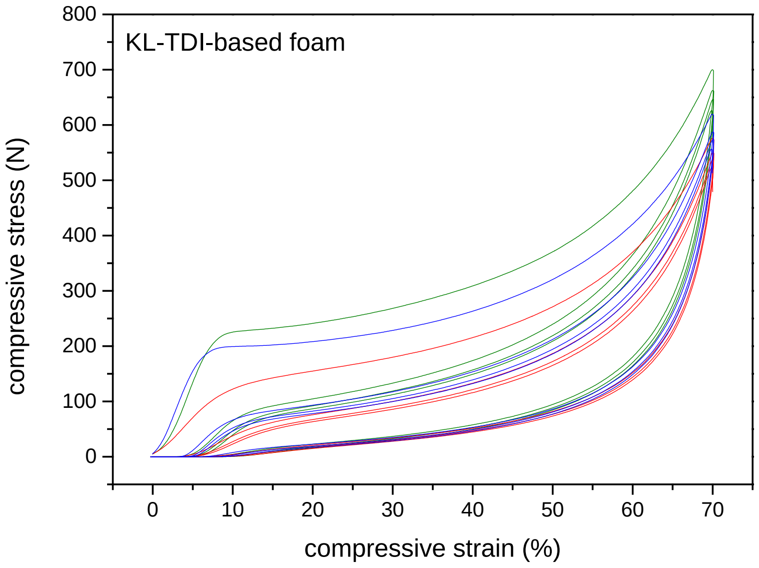
KL-TDI-based foam
compressive stress (N)
compressive strain (%)
800
700
600
500
400
300
200
100
0
0
10
20
30
40
50
60
70

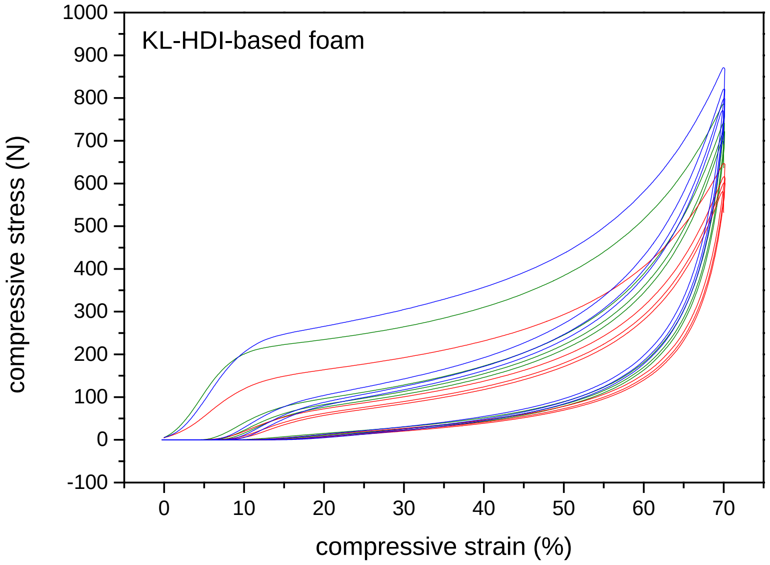

7. References

Adamo, C. (1999). Toward reliable density functional methods without adjustable parameters: The PBE0 model, J. Chem. Phys. 110, 6158.

DIN ISO 3386-1:2015-10 (2015). "Polymeric materials, cellular flexible - Determination of stress-strain characteristics in compression - Part 1: Low-density materials.".

DIN ISO 14896:2009 (2009). "Kunststoffe - Polyurethanstoffe - Bestimmung des Isocyanatanteils (ISO 14896:2009)", in: *Ref. Nr. EN ISO 14896:2009D.* European Committee for Standardization).

Granata, A., and Argyropoulos, D.S. (1995). 2-Chloro-4, 4, 5, 5-tetramethyl-1, 3, 2-dioxaphospholane, a reagent for the accurate determination of the uncondensed and condensed phenolic moieties in lignins. *Journal of Agricultural and Food Chemistry* 43**,** 1538-1544.

Larsen, A. H.; Mortensen, J. J.; Blomqvist, J.; Castelli, I. E.; Christensen, R.; Dułak, M.; Friis, J.; Groves, M. N.; Hammer, B.; Hargus, C. (2017). The atomic simulation environment — a Python library for working with atoms, *J. Phys.: Condens. Matter,* 29, 273002.

Mortensen, J. J.; Hansen, L. B.; Jacobsen, K. W. (2005). Real-space grid implementation of the projector augmented wave method, Phys. Rev. B 71, 035109.

Perdew, J. P.; Burke, K.; Ernzerhof, M. (1996). Generalized Gradient Approximation Made Simple, *Phys. Rev. Lett.* 77, 3865.

V., D.D.I.F.N.E. (2009). "Pla*stics - Polyurethane raw materials - Determination of isocyanate content", in: ISO 14896:2009. (ed.) DIN.).*

Stephens, P. J.; Devlin, F. J.; Chabalowski, C. F.; Frisch, M. J. (1994). Ab Initio Calculation of Vibrational Absorption and Circular Dichroism Spectra Using Density Functional Force Fields, J. Phys. Chem.*, 98, 11623–11627.*

Zawadzki, M., and Ragauskas, A. (2001). N-hydroxy compounds as new internal standards for the 31P-NMR determination of lignin hydroxy functional groups. *Holzforschung* 55**,** 283-285.
